# Intrinsic activation of cell growth and differentiation in *ex vivo* cultured human hair follicles by a transient endogenous production of ROS

**DOI:** 10.1038/s41598-019-39992-8

**Published:** 2019-03-14

**Authors:** María I. Calvo-Sánchez, Sandra Fernández-Martos, Juan José Montoya, Jesús Espada

**Affiliations:** 1 Ramón y Cajal Institute for Health Research (IRYCIS), Ramón y Cajal University Hospital, Colmenar Viejo Road Km. 9,100, 28034 Madrid, Spain; 2 Department of Radiology, Rehabilitation & Physiotherapy. Faculty of Medicine, Complutense University, Madrid, 28040 Spain; 3 Centro Integrativo de Biología y Química Aplicada (CIBQA), Universidad Bernardo O´Higgins, General Gana 1780, Santiago, 8370854 Chile; 4 Instituto de Investigaciones Biosanitarias. Facultad de Ciencias Experimentales, Universidad Francisco de Vitoria, 28223 Pozuelo de Alarcón, Madrid Spain

**Keywords:** Growth factor signalling, Cell proliferation

## Abstract

The emerging variety of signalling roles for ROS in eukaryotic cells and tissues is currently a matter of intense research. Here we make use of *ex vivo* cultured single human hair follicles as an experimental model to demonstrate that a transient production of non-lethal endogenous ROS levels in these mini-organs promotes efficiently the entry into the growth phase (anagen). The stimulatory process implicates the specific activation of the hair follicle stem cell niche, encompassing the induction of stem cell differentiation markers (Ck15), overall cell proliferation and sustained growth of the tissue associated with expression of gen targets (Ccnd1) concomitant with the inhibition of Wnt signaling antagonists and repressors (Dkk1, Gsk3β) of Wnt signaling. As a whole, this observation indicates that, once activated, ROS signalling is an intrinsic mechanism regulating the hair follicle stem cell niche independently of any external signal.

## Introduction

The potential signalling roles in eukaryotic cells of a dedicated and local production of non-lethal ROS levels is currently a matter of intense research. The deleterious effects of an incidental ROS accumulation inside cells and tissues, mostly due to a leaky electron transport chain in the mitochondria, are widely characterized. Deregulated ROS production can promote the accumulative oxidation and further functional inactivation of several cell components, including lipids, amino acids, enzyme co-factors and the DNA molecule^1–3^. These oxidative events commonly result in the irreversible activation of cell death mechanisms^4,5^. Not surprisingly, ROS-dependent damage has been associated at a systemic level with several human diseases and with the ageing process^1–3,5,6^.

In the last years, different results using cultured eukaryotic cells have pointed out that ROS can be directly involved in the regulation of key processes such as proliferation, differentiation and motility^3,4,7^, underpinning the notion of these molecules as true biochemical signalling effectors. In support of this new paradigm in redox biology, organotypic and animal experimental models have been also used trying to establish the physiological roles of ROS *in vivo*. In this regard, it has been shown that the differentiation program of hematopoietic progenitors in *Drosophila* is associated with local changes in ROS concentrations^8^. In zebrafish, wound induction triggers the formation of a transient ROS gradient in the tissue and a similar event occurs during tail regeneration in *Xenopus* tadpoles^9^. In mammals, the exogenous administration of hydrogen peroxide activates the self-renewal and differentiation programs of neural stem cells in a neurosphere model^10^. In this context, it has been proposed an active role for ROS during vertebrate embryo development^11,12^. Interestingly, a transient and differential ROS production has been associated also with the deregulation of intestinal stem cell function during colorectal cancer initiation^13^.

In mouse skin, genetic impairment of mitochondrial activity by a conditional deletion of the mitochondrial Transcription Factor A (TFAM) locus results in defective hair follicle growth^14^, indirectly suggesting a role for ROS in the modulation of hair follicle stem cells. More recently, we have provided a straightforward demonstration of the physiological roles of ROS *in vivo* in the regulation of skin homeostasis and regeneration and of the hair follicle stem cell niche activity^15,16^. Using Protoporphyrin IX as an endogenous photosensitizer and subsequent irradiation of the tissue with red light to promote a local, spatially restricted photodynamic effect, we have reported that a transient activation of non-lethal endogenous ROS levels activates the hair follicle stem cell niche, promoting wound healing and hair growth in a ROS-dependent process. In a step forward, our aim here, using human hair follicles growth *ex vivo* as experimental model, was to provide a concise report supporting the notion that the ROS-dependent activation of the hair follicle stem cell niche is an intrinsic event that does not depend on signals from the surrounding tissue.

## Results and Discussion

**Figure 1:**
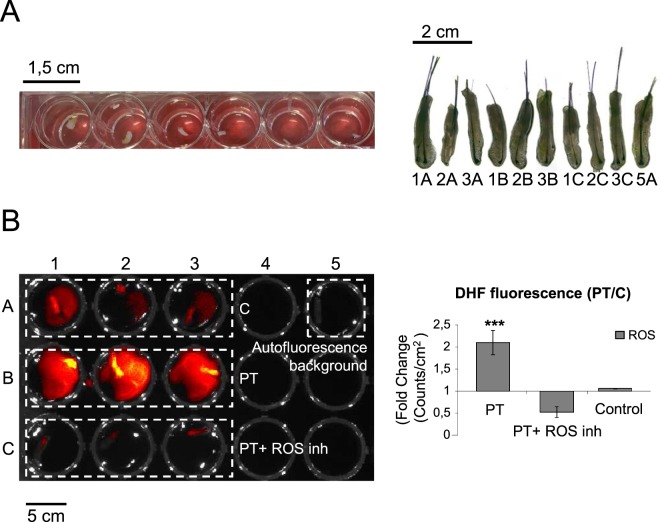
Transient activation of endogenous ROS production in human hair follicles by a PpIX-dependent photodynamic treatment. (**A**) Brightfield microscope images of representative hair follicles growing in 24-well plates (left panel) and the specific series of hair follicles used in the experiment shown in (**B**) at day 0 before ROS activation, indicating the 24-well location code for each sample (right panel). (**B**) Imaging of endogenous ROS production in human hair follicles grown *ex vivo* after a Protoporphyrin IX-dependent photodynamic treatment (incubation for 4 h with 0,1 mM with Protoporphyrin IX precursor m-ALA and subsequent irradiation with 634 nm red light, total light dose of 3,72 J/cm^2^; PT) or after PT in the presence of a ROS scavenger cocktail (PT + inh) (3 mM N-acetil-L-cysteine and 100 µM Ascorbic Acid). Control samples were irradiated solely with red light. ROS was detected using an IVIS-Lumina system using 2′,7′-dichlorodihydrofluorescein diacetate (DHF-DA) as a generic fluorescent ROS reporter. Essentially the same results were obtained with singlet oxygen Sensor Green reagent. Fluorescence intensity was recorded as Counts (photons/sec/cm^2^). Results are representative of three different experiments (n ≥ 3 samples per condition in each experiment). (**C**) Quantification of ROS dependent fluorescence under the described experimental conditions. The ratio between the counts obtained from Photodynamic Treatment (PT) and Control groups or Photodynamic treatment with ROS scavengers (PT + inh) and Control was represented. The mean +/− SD values of at least three independent experiments (n ≥ 3 samples per condition in each experiment) are represented. ***significant P < 0.001.

**Figure 2:**
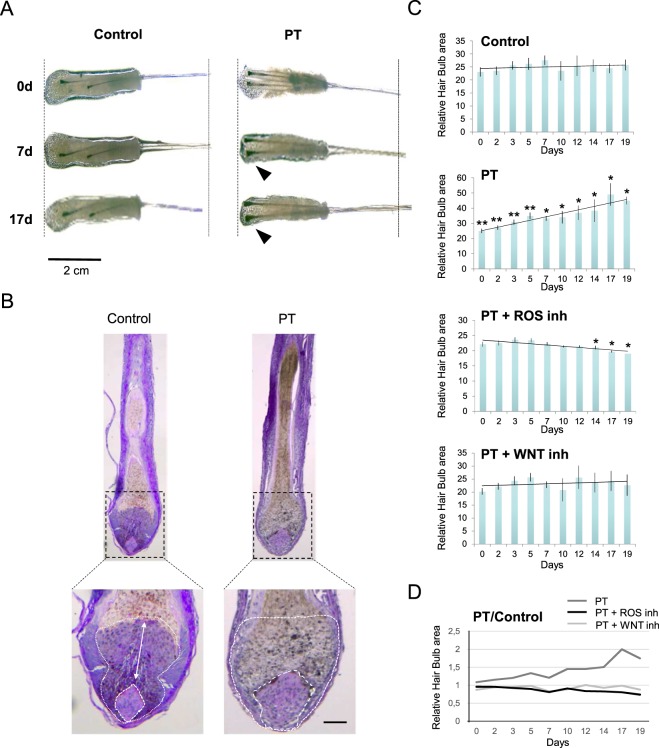
Induction of a sustained WNT-dependent thickening of the hair bulb/dermal papilla region of human hair follicles by a PpIX-dependent non-lethal production of endogenous ROS. (**A**) Representative images of hair bulb thickening at 0, 7 and 19 days after PpIX-dependent Photodynamic Treatment (PT). A local non-lethal ROS production induces a significant and sustained increase in the whole hair bulb area of human hair follicles growth *ex vivo* after PT as compared to Control conditions. Note that PT hair follicles are still growing 19 days after the treatment. Results are representative of at least 50 different hair follicles. (**B**) Representative histological sections stained with Masson´s trichrome of Control or PT human hair follicles grown *ex vivo* after 7 days in culture. Note the enlargement of the dermal papilla (large dotted line) and hair matrix (small dotted line) regions in PT samples. Results shown are representative of at least 10 hair follicles in three independent experiments. Bar: 50 µm. (**C**) Time course quantification of hair bulb area after red light irradiation (light Control), PT dependent ROS production (PT condition), and PT in the presence of a specific WNT signalling pathway inhibitor (PT + WNT inh) or ROS scavengers (PT + ROS inh). A local, PpIX-dependent non-lethal ROS production induces a sustained and strongly significant increase of the hair bulb area after PT as compared to Control conditions, that is abolished by the WNT inhibitor PNU-74654 [40 µM], or the ROS scavengers AA (60 ng/µl) and NAC (480 ng/µl). Results are representative of at least 18 hair follicle units per condition. The mean +/− SD of n ≥ 10 for each experimental condition is represented and T-test was used for statistical analysis. **significant P ≤ 0.05; *significant P ≤ 0.1. (**D**) Time course representation of the ratios between the mean area of treatment (PT, PT + WNT inh, PT + ROS inh) relative the Control conditions quantified in (**C**).

Here we have used as experimental system human follicular units (FUs) grown *ex vivo* typically containing one or two individualized hair follicles in the late resting (telogen)/early growing (anagen) phase, embedded in a remnant matrix of fatty and dermal tissues (Fig. 1A). In our experience, maintenance of this remnant matrix promotes optimal hair follicle survival up to day 7–9 in basal conditions and the presence of one or two hair follicles in the FU are virtually equivalent experimental conditions. First, we verified that treatment of resting (telogen phase) human hair follicles with low concentrations of 5 mALA, a precursor of PpIX, and subsequent irradiation with a moderate red-light dose promoted a transient ROS burst in the cultured mini-organs (Fig. 1B). In the experimental conditions described here, the resultant transient peak of ROS production induced by these photodynamic effect was found non-lethal, in agreement with our previous results in epidermal cells and in mouse skin^15,16^. We further found that the activation of a transient, non-lethal ROS production induced by a PpIX-dependent photodynamic treatment (PT) was sufficient to induce a rapid and evident swelling of the hair bulb region of treated hair follicles as compared to Control partners, suggesting the entrance into the growing (anagen) phase (Fig. 2A). A detailed histological analysis showed a significant increase in the cellular mass of the dermal papilla and the whole hair bulb region 7 days after PT (Fig. 2B). A time course image-based quantitative analysis in *ex vivo* grown hair follicles further confirmed the significant increase of the hair bulb area induced by PT. A steady and continuous growth was observed in treated hair follicles for more than 18 days, while Control samples did not show significant changes in the hair bulb area (Fig. 2C,D). All these stimulatory events, as well as the transient ROS production induced by the photodynamic treatment, were completely abolished by a cocktail of antioxidant compounds (Figs 1 and 2), indicating the direct implication of ROS production in the subjacent molecular mechanisms. Interestingly, the ROS-dependent induction of hair bulb growth was also strongly inhibited in the presence of the specific WNT inhibitor PNU-74654 (Fig. 2C,D), that specifically prevents the binding of β-catenin to LEF/TCF factors, pointing out the implication of the WNT/β-catenin signaling pathway in this process.

**Figure 3:**
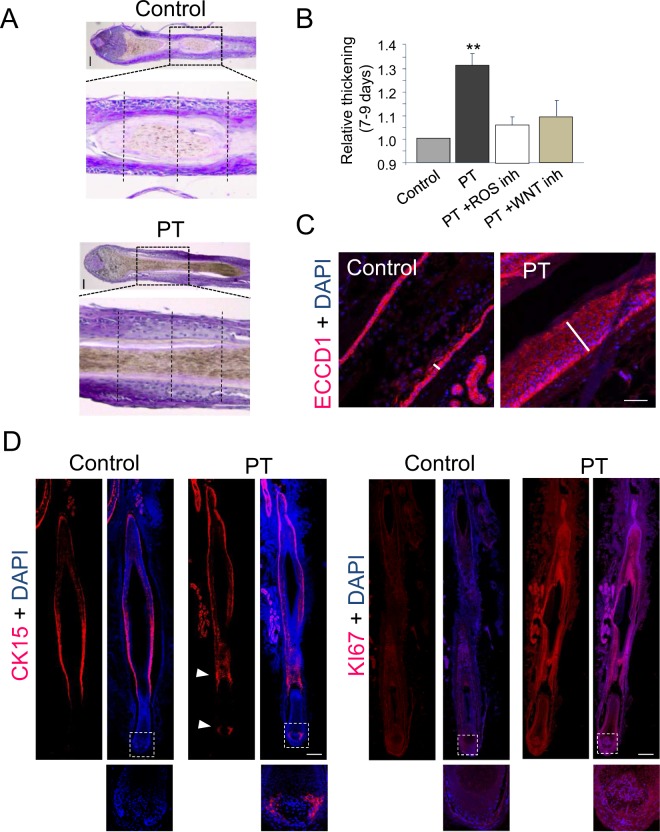
Induction of WNT-dependent suprabulbar fibre thickening of human hair follicles by a PpIX-dependent non-lethal production of endogenous ROS associated with the expression of proliferation and stem cells markers. (**A**) Representative histological sections stained with Masson´s trichrome of human hair follicles grown *ex vivo* of light Control or Protoporphyrin IX-dependent photodynamic treatment (PT) samples after 7 days in culture (same as in Fig. 2 for comparative purposes) showing the thickness of the suprabulbar region. Results shown are representative of at least three hair follicles in three independent experiments. Right panels show the magnification of regions indicated in left panels. Doted lines are representative examples showing proximal, medial and distal locations where suprabulbar hair fiber transversal length was measured. Bar: 50 µm. (**B**) Quantification of suprabulbar hair thickness showing a statistically significant increase in PT as compared to Control samples that is abolished by the WNT inhibitor PNU-74654 [40 µM], or the ROS scavengers AA (60 ng/µl) and NAC (480 ng/µl). The mean +/− SD of the main suprabulbar thickness (mean of proximal, medial and distal length measurement in each hair follicle) in n = 3 samples for each experimental condition was represented and T-test was used for statistical analysis. (**C**) Representative confocal microscopy images (maximum projections) of the immunolocalization of the E-Cadherin cell-cell adhesion protein (ECCDN1) in the suprabulbar region in histological sections of human hair follicles grown *ex vivo* 7 days after red light irradiation (light Control) or PT samples. DAPI was used as chromatin counterstain and is shown in merged channels. Note the significant increase in the number of ECCD1 positive cells in outer and inner root sheath epidermal layers (bars). Results are representative of three different experiments, n ≥ 3 samples per condition in each experiment. Scale bars: 50 µm. (**D**) Representative confocal microscopy images (maximum projections) of the immunolocalization of the Cytokeratin 15 (CK15) stem cell marker and the Ki67 cell proliferation marker in histological sections of human hair follicles grown *ex vivo* 7 days after red light irradiation (light Control) or PT. DAPI was used as chromatin counterstain and is shown in merged channels. Results are representative of three different experiments, n ≥ 3 samples per condition in each experiment. Lower panels shown the magnification of hair bulb regions indicated in upper panels. Scale bars: 100 µm.

We next investigated whether the hair bulb growth induced by a non-lethal ROS production was also associated with cell proliferation and thickening of the suprabulbar hair fiber. Morphological analysis of histological section showed a noticeable increase in the number of cell layers and in the length of the hair fiber 7 days after PT, as compared to Control samples (Fig. 3A). Quantification of suprabulbar hair fiber transversal length at day 7 showed a statistically significant thickness increase induced by PT that was abrogated by ROS scavengers and the PNU-74654 WNT inhibitor (Fig. 3B). The immunolocalization of the E-cadherin homotypic cell-cell adhesion receptor further confirmed the strong increase in hair suprabulbar fiber thickness and in the number of epithelial cell layers in the inner and outer root sheets 7 days after PT (Fig. 3C). The proliferation and mobilization of skin stem cell progenitors was monitored by the expression of the specific CK15 marker in the tissue (Fig. 3D), showing that an activation of proliferation and differentiation programs occurred in the hair follicle stem cell niche in response to ROS signaling. The overall activation of cell proliferation induced by a transient production of ROS in the hair follicle was corroborated by the immunolocalization of the KI67 marker, showing a significant increase in expression levels in the tissue 7 days after PT as compared to Control samples (Fig. 3D).

**Figure 4:**
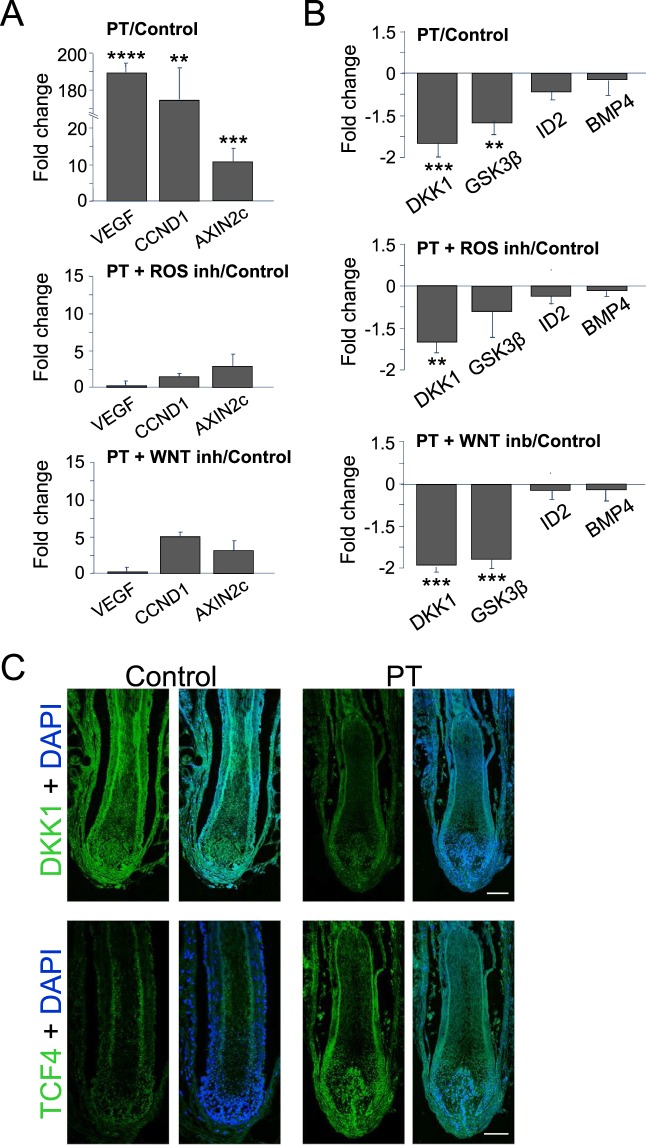
Transcriptional activation of WNT signalling gene targets and of hair follicle growth promoting factors in human hair follicles induced by a PpIX-dependent non-lethal production of endogenous ROS. (**A**) Gene expression analysis of selected WNT signalling transcriptional targets in the skin (CCDN1, AXIN2c) and of a key human hair follicle growth promoting factor (VEGF) in human hair follicles growth *ex vivo*. Note the strong expression of these gene targets 7 days after a transient, non-lethal ROS production in the tissue induced by a Protoporphyrin IX-dependent photodynamic treatment (PT), that is abolished by the WNT inhibitor PNU-74654 [40 µM], or the ROS scavengers AA (60 ng/µl) and NAC (480 ng/µl). (**B**) Gene expression analysis of WNT signalling inhibitors/antagonists (GSK3β, DKK1) and of transcriptional effectors (BMP4) and targets (ID2) of BMP/Smad signalling. Note that GSK3β and DKK1 expression is significantly downregulated by PT and this downregulation is not modulated by WNT or ROS inhibition. Note also that the expression of BMP4 or ID2 is not affected by PT. In (**A**,**B**), gene expression values were quantified by qRT-PCR, fold changes were calculated as the ratio between means of 2^−ΔCt^ values normalized to 18S rRNA, and the means of fold changes (relative to Control) +/− SE of n ≥ 4 samples for each experimental condition were represented. T-test was used for statistical analysis. ****significant P ≤ 0.0005; ***significant P ≤ 0.001, **significant P ≤ 0.05; *significant P ≤ 0.1. (**C**) Representative confocal microscopy images (maximum projections) of the immunolocalization of the WNT signalling antagonist DKK1 and the WNT signalling dependent transcription factor TCF4 in histological sections of human hair follicles grown *ex vivo* 7 days after red light irradiation (light Control) or PT. DAPI was used as chromatin counterstain and is shown in merged channels. Results are representative of two different experiments, n ≥ 3 samples per condition in each experiment. Scale bars: 100 µm.

Two master signaling networks control for the most part the hair follicle growth cycle; WNT/β-catenin signaling regulates the activation of the stem cell niche and the entrance into the growing (anagen) phase while BMP/Smad signaling is implicated in the maintenance of the quiescence state in the hair follicle stem cell niche and the entrance into the resting (telogen) phase^17^. We have shown above that WNT signaling is implicated in the ROS-dependent stimulation of human hair follicles grown *ex vivo* (Figs 2 and 3). A quantitative analysis of gene expression further indicated that a transient production of non-lethal ROS levels in the tissue strongly activated the transcription of key WNT signaling targets in the skin (CCND1, AXIN2c) and of a factor (VEGF) that acts coordinately with the WNT pathway to control hair growth and follicle size in the mouse^18^ and to induce dermal papilla cell proliferation in human hair follicles^19^ (Fig. 4A). Notably, the expression activation of these gene targets was completely abrogated by both ROS scavenging and WNT signaling inhibition (Fig. 4A). In addition, a transient production of non-lethal ROS levels in human hair follicles induced a significant downregulation WNT signaling antagonists and repressors (DKK1, GSK3β), while no alterations were observed in the expression of BMP/Smad signaling gene targets (ID2) or effectors (BMP4) (Fig. 4B). Interestingly, none of these expression patterns where affected by either ROS scavenging or WNT inhibition (Fig. 4B). DKK1 gene expression pattern was confirmed by immunolocalization experiments (Fig. 4C). Finally, we also analyzed the protein expression pattern of the WNT signaling-related transcription factor TCF4 in human hair follicles, and we found that this protein was significantly expressed in the dermal papilla 7 days after PT as compared to Control samples (Fig. 4C), in agreement with previous results^20,21^. These observations suggest that functional ROS signaling may be involved in the modulation of the WNT, but not BMP/Smad, signaling network in the hair follicle.

As a whole, the results reported here point out the physiological ROS signaling as a cell autonomous process that, once triggered, does not require any further external cue to proceed. In mammalian skin, the hair follicle is a complex mini organ that alternates growth, regression and resting phases depending on external signals coming from the surrounding environment^17^. For the most part, these external signals regulate the activity of the hair follicle stem cell niche. As a consequence, the continuous growth of hair follicles *ex vivo* requires a mixture of growth factors added externally to maintain an active proliferative state that, in human follicles, last for no more than 7–10 days as a rule, at which time the whole structure enters in regression (catagen) phase. Here we provide evidence indicating that activation of a transient production of non-lethal levels of endogenous ROS promotes a continuous growth and differentiation of human hair follicles *ex vivo* for more than 20 days associated with the activation of hair follicle stem cells. In this sense, the results shown here strongly support an active role for ROS in the functional regulation of stem cell niches, in agreement with previous reports. In this particular experimental model, ROS signaling is able to switch on the main pathway implicated in the activation of the hair follicle stem cell niche, namely Wnt/β-catenin, but does not affect BMP/Smad signaling, responsible for the resting phase of this niche.

The underlaying molecular mechanism of the ROS-dependent activation of the hair follicle stem cell niche deserves further investigation and constitutes and exciting area of research. Major molecular targets of ROS signaling at cell/tissue functional level are redox sensing enzymes, mostly protein kinases and phosphatases harboring critical sulphur-containing amino acids in the catalytic domain susceptible of reversible functional oxidation^22,23^. In this sense, the Src kinase is a prototypic redox sensing enzyme^22,24^ and we have previously shown that this enzyme is specifically activated in mouse skin after a transient ROS stimulus that promotes hair growth and wound healing^15^. It has been also reported that SRC and Wnt signaling act together in critical aspects during *C*. *elegans* embryo development^25^, pointing out a potential interconnection node between ROS and Wnt signaling. Kinases and phosphatases of the ERK/MAPK signaling network are also a proposed key target of ROS^23^. Interestingly, a cross-talk, either in positive or negative feedback settings, between ERK/MAPK and Wnt/β-catenin pathways has been reported in different experimental models, including the skin^26^. Overall, although the specific role for ROS in hair follicle dynamics has not investigated in deep in this work, the reported results provide further evidence of potential physiological roles for ROS in the functional regulation of complex biological structures, in this case the human hair follicle.

## Materials and Methods

All methods were performed in accordance with all relevant institutional and EU experimental and ethical guidelines.

### *Ex vivo* culture of human hair follicles

Human hair follicles were obtained from human scalp samples taken from the occipital skin of volunteer donors during a hair transplant procedure. Eligible patients provided written informed consent, and the Ethical committee of the Ramon y Cajal University Hospital approved this procedure. Follicular units (FUs) in the resting (telogen) phase, typically containing one or two hair follicles and surrounding fatty and dermal tissue remnants, were dissected and selected by expert trichologists at the Ramón y Cajal Hospital Dermatology Service using standard morphological criteria. FUs were grown in Williams E medium (Sigma) supplemented with 10x Penicilin/Streptomycin, 10x Amphotericin B, and 2 mM L-Glutamin (all from Gibco), 5 µg/ml Insulin, 5 µg/ml transferring, 20 pM T3 hormone, 0.083 µg/ml cholera toxin and 0.4 µg/ml hydrocortisone at 37 °C in a 5% CO_2_ humidified atmosphere. Adherent tissue remnants in FUs were maintained throughout the *ex vivo* growing process to improve hair follicle viability in basal conditions. FUs from the same individual and body location were used in each experimental series.

### ROS production

A modified (patented for all commercial purposes) PpIX-dependent photodynamic treatment was used to activate a transient production of non-lethal endogenous ROS levels in human hair follicles *in vivo*. To this end, FUs were incubated for 4 hours with 0,1–1 mM of MAL (Sigma) in complete Williams E medium, and then irradiated 10 min with a red light emitting diode source with an emission peak at 634 nm bandwidth, with a total light dose of 3.72 J/cm^2^. Light controls with no MAL were included in all experimental designs. PT + inh condition was carried out adding the ROS scavengers N-acetil-L-Cysteine and Ascorbic Acid (NAC and AA, both from Sigma-Aldrich) at a final concentration of 3 mM and 100 µM, respectively, 30 min before red light exposure, to impair the effects associated to ROS. After treatments, fresh medium was added. In order to inhibit WNT signaling activity, the specific inhibitor PNU-74654 (Santa Cruz Biotech) was used at a final concentration of 40 µM, diluted in complete Williams medium E. PNU-74654 was initially added to the medium at the same that MAL and was maintained until the end of experiments, replacing every day with fresh medium containing the inhibitor.

### ROS quantification

The determination and quantification of ROS production was carried out using the fluorescent probes 2′,7-Dichlorodihydrofluorescein diacetate (DHF-DA, Sigma-Aldrich) and singlet oxygen Sensor Green reagent (Thermo Fisher). The probes were diluted in Williams E medium during MAL incubation, at a final concentration of 10 µM, 30 min before red light irradiation. 30 minutes after red light irradiation, hair follicles were washed twice in saline solution, placed into a black multiwell plate with saline solution, and the plate was transferred into an IVIS device (IVIS Lumina 2 imagen system, xenogeny). ROS production was evaluated by measuring DCF signal selecting the adequate filters (445–490 nm for the excitation band, 515–575 nm for the emission band).

Optical acquisition settings were adjusted to obtain a signal level of 600 to 60000 relative lux counts. To this end, oxidized H2-DCF-DA (DCF) molecules were excited during 1 second of exposition time, with an aperture (amount of light collected, F/Stop) of 8. In order to improve the signal to noise ratio for read noise, Small Binning was selected. Data was collected as counts (photons/sec/cm^2^) using Living Image software v2.50 (Xenogen). The minimum colour scale was adjusted to the negative control (FU without DHF-DA), and the maximum colour scale to the PT area. The regions of interest (ROI) were adjusted to wells’ size, and the fluorescence measurements of each ROI were exported in excel format. The ratio between the total counts and the selected area was calculated and compared as fold change between PT and control, or PT + inh and control. T-test was used for statistical analysis.

### Histological analysis

For histological analysis, FUs were fixed in 3.7% aqueous pH-neutral formaldehyde and embedded in paraffin using standard procedures. Typically, 18–22, 6 µm thick, longitudinal tissue sections were obtained for each FU. As a rule, the most 6–4 central sections, encompassing as much as much as possible the full length of, at least, the suprabulbar fibre and hair bulb regions, were used for comparative analysis. Remnant tissue sections were used to implement immunological procedures (see below). To analyse morphological changes at tissue level, histologically equivalent sections were evaluated by Masson’s trichrome staining. Bright field image acquisition was performed using a Nikon Eclipse Ci coupled to a Jenoptik PROGRES GRYPHAX^®^ SUBRA Super HD camera and suited Version 1.1.8.153 image software pack.

### Quantification of hair bulb area and suprabulbar fibre thickening

For the evaluation of significant morphological changes *in vivo* in whole hair follicles, high resolution images of FUs growing in 24-well plates were acquired at time 0 after PT, and every 24 hours onwards for a minimum of 7 and a maximum of 19 days, depending on experimental settings. Image acquisition was performed using a Nikon Eclipse Ci LED-fluorescence microscope using a 2x objective and a 0.55x Reduction Lens adapter coupled to a Jenoptik PROGRES GRYPHAX^®^ SUBRA Super HD camera. Total hair bulb area in high resolution images of whole hair follicles was spotted and quantified using suited free FIJI software packs (https://fiji.sc/). The mean +/− SD of hair bulb measurements in n ≥ 12 samples for each experimental condition was represented and T-test was used for statistical analysis. As adherent tissue remnants in growing FUs preclude a precise imaging *in vivo* of the hair fibre, equivalent histological sections of the medial hair follicle region were used for the evaluation of suprabulbar fibre region thickening. Hair follicles at day 7 were selected for this set of measurements. High resolution images of Masson´s trichrome or DAPI stained sections acquired in a Nikon Eclipse Ci LED-fluorescence microscope were used to quantify the transversal length of the hair fibre in three separate locations of the suprabulbar region, proximal, medial and distal, for each hair follicle (see Fig. 3A). The mean of these three measurements was used as the main hair follicle suprabulbar fibre thickness value. The mean +/− SD of suprabulbar thickness in n = 3 samples for each experimental condition was represented and T-test was used for statistical analysis.

### RNA extraction and gene expression analysis

Total RNA of at least 4 FUs in each experimental group was extracted 7 days after treatments, using RNeasy micro kit (Qiagen). RNA was normalized with respect to the number of FUs in each experimental condition, and then was converted into cDNA using FastGene Scriptase II cDNA Kit (Nippon genetics). qRT-PCR data was analysed using a comparative CT method, using 18S ribosomal RNA expression as an internal control. Gene expression fold changes were represented as the ratio between means of 2^−ΔCt^ values of MAL FUs and Light control FUs mean values. Gene targets and primers (reading 5′–3′) used for amplifications were as follows:

CCND1: Fw- ACGAAGGTCTGCGCGTGTT; Rev- CCGCTGGCCATGAACTACCT

GSK3β: Fw- AACTGCCCGACTAACAACAC; Rev- ATTGGTCTGTCCACGGTCTC

VEGF: Fw- GAGATGTCCCTGGAAGAACACA; Rev- GAGTGGGATGGGTGATGTCAG

ID2:Fw- GCTATACAACATGAACGACTGCT; Rev- AATAGTGGGATGCGAGTCCAG

AXIN2c: Fw- GGTGTTTGAGGAGATCTGGG; Rev- TGCTCACAGCCAAGACAGTT

BMP4: Fw- AAAGTCGCCGAGATTCAGGG; Rev- GACGGCACTCTTGCTAGGC

DKK1: Fw- CCTTGAACTCGGTTCTCAATTCC; Rev- CAATGGTCTGGTACTTATTCCCG

18S: Fw- CGGCTACCACATCCAAGGAA; Rev- GCTGGAATTACCGCGGCT

### Protein immunolocalization

To determine target protein localization and expression patterns in hair follicles, histological tissues sections were used. At least one FU in each experimental group was fixed in 3.7% aqueous formaldehyde and embedded in paraffin as described above. Antigen retrieval was performed using 10 mM Citrate Buffer in hydrated sections following standard procedures. Primary antibodies, including anti-Cytokeratin 15 (CK15, clon EPR16Y, Abcam) and anti-KI67 (clon SP6, Abcam), anti-E-Cadherin (ECCD1; clon 24E10, Cell Signalling), anti-TCF4 (clon D4, Santa Cruz Biotech) and anti-DKK1 (clon B7, Santa Cruz Biotech), were incubated over night at 4 °C in a wet chamber, extensively washed with PBS, incubated for 1 hour at room temperature with appropriate secondary fluorescence-labelled antibodies and mounted in DAPI (100 ng/ml)-containing Vectashield. Confocal images were obtained in Leica TCS SP5 AOBS spectral confocal microscope and processed using the FIJI software.
