# Publisher Correction: Intrinsic activation of cell growth and differentiation in *ex vivo* cultured human hair follicles by a transient endogenous production of ROS

**DOI:** 10.1038/s41598-019-55123-9

**Published:** 2019-12-04

**Authors:** María I. Calvo-Sánchez, Sandra Fernández-Martos, Juan José Montoya, Jesús Espada

**Affiliations:** 1 Ramón y Cajal Institute for Health Research (IRYCIS), Ramón y Cajal University Hospital, Colmenar Viejo Road Km. 9,100, 28034 Madrid, Spain; 2 Department of Radiology, Rehabilitation & Physiotherapy. Faculty of Medicine, Complutense University, Madrid, 28040 Spain; 3 Centro Integrativo de Biología y Química Aplicada (CIBQA), Universidad Bernardo O´Higgins, General Gana 1780, Santiago, 8370854 Chile; 4 Instituto de Investigaciones Biosanitarias. Facultad de Ciencias Experimentales, Universidad Francisco de Vitoria, 28223, Pozuelo de Alarcón, Madrid, Spain

Correction to: *Scientific Reports* 10.1038/s41598-019-39992-8, published online 14 March 2019

This Article contains errors.

In the Results and Discussion section where,

“First, we verified that treatment of resting (telogen phase) human hair follicles with low concentrations of 5 mALA, a precursor of PpIX, and subsequent irradiation with a moderate red-light dose promoted a transient ROS burst in the cultured mini-organs (Fig. 1B).”

should read:

“First, we verified that treatment of late resting (telogen)/early growing (anagen) phase human hair follicles with low concentrations of 5 mALA, a precursor of PpIX, and subsequent irradiation with a moderate red-light dose promoted a transient ROS burst in the cultured mini-organs (Fig. 1B).”

Additionally, in the Materials and Methods section under subheading ‘*Ex vivo* culture of human hair follicles’ where,

“Follicular units (FUs) in the resting (telogen) phase, typically containing one or two hair follicles and surrounding fatty and dermal tissue remnants, were dissected and selected by expert trichologists at the Ramón y Cajal Hospital Dermatology Service using standard morphological criteria.”

should read:

“Follicular units (FUs) in late resting (telogen)/early growing (anagen) phase, typically containing one or two hair follicles and surrounding fatty and dermal tissue remnants, were dissected and selected by expert trichologists at the Ramón y Cajal Hospital Dermatology Service using standard morphological criteria.”

Additionally, in Figure 3A the panels have been inadvertently rotated. The correct Figure 3 appears below as Figure 1.

**Figure 1:**
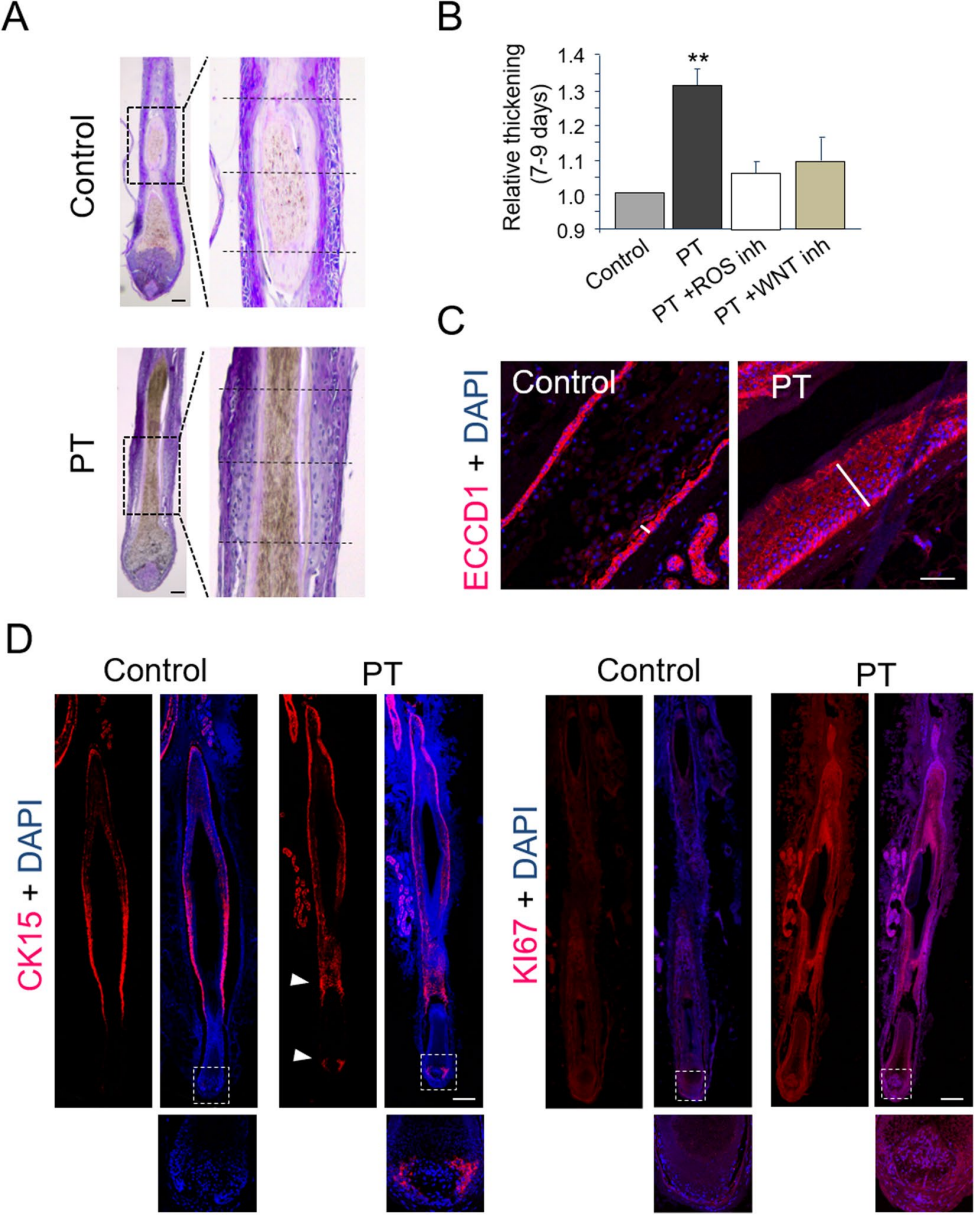
.

